# Online Information-Seeking About Potential Breast Cancer Symptoms: Capturing Online Behavior With an Internet Browsing Tracking Tool

**DOI:** 10.2196/12400

**Published:** 2019-02-06

**Authors:** Afrodita Marcu, Cecile Muller, Emma Ream, Katriina L Whitaker

**Affiliations:** 1 School of Health Sciences Faculty of Health & Medical Sciences University of Surrey Guildford United Kingdom; 2 School of Psychology Faculty of Health & Medical Sciences University of Surrey Guildford United Kingdom

**Keywords:** breast cancer, health information, internet search, online information seeking

## Abstract

**Background:**

People engage in health information-seeking online when experiencing unusual or unfamiliar bodily changes. It is not well understood how people consult the internet for health information after the onset of unfamiliar symptoms and before receiving a potential diagnosis and how online information-seeking can help people appraise their symptoms. This lack of evidence may be partly due to methodological limitations in capturing in real time the online information-seeking process.

**Objective:**

We explored women’s symptom attribution and online health information-seeking in response to a hypothetical and unfamiliar breast change suggestive of cancer (nipple rash). We also aimed to establish the feasibility of capturing in real time the online information-seeking process with a tool designed to track participant online searches and visited websites, the Vizzata browser tracker.

**Methods:**

An online survey was completed by 56 cancer-free women (mean age 60.34 [SD 7.73] years) responding to a scenario asking them to imagine noticing a red scaly rash on the nipple. Participants were asked to make symptom attributions when presented with the scenario (T1) and again after seeking information online (T2). The online tracking tool, embedded in the survey, was used to capture in real time participant search terms and accessed websites.

**Results:**

The tracking tool captured the search terms and accessed websites of most of the participants (46/56, 82%). For the rest (10/56, 18%), there was evidence of engagement in online information-seeking (eg, medical terminology and cancer attribution at T2) despite their searching activity not being recorded. A total of 25 participants considered cancer as a potential cause for the nipple rash at T1, yet only one of these used cancer as a search term. Most participants (40/46, 87%) used rash-related search terms, particularly nipple rash and rash on nipple. The majority (41/46, 89%) accessed websites containing breast cancer information, with the National Health Service webpage “Paget disease of the nipple” being the most visited one. At T2, after engaging in the internet search task, more participants attributed the nipple rash to breast cancer than at T1 (37/46, 66% vs 25/46, 45%), although a small number of participants (6/46) changed from making a cancer attribution at T1 to a noncancer one at T2.

**Conclusions:**

Making a cancer attribution for an unfamiliar breast change did not necessarily translate into cancer-termed searches. Equally, not all internet searches led to a cancer attribution. The findings suggest that online information-seeking may not necessarily help women who experience unfamiliar breast cancer symptoms understand their condition. Despite some technical issues, this study showed that it is feasible to use an online browser tracking tool to capture in real time information-seeking about unfamiliar symptoms.

## Introduction

Seeking health information online is a ubiquitous activity, enabled by advances in Web 2.0 design (which includes user-generated content), developments in search engines, proliferation of social media, and wide ownership of mobile phones, tablets, and computers [1]. The Google search engine is commonly used in online health searches [2]. Of the world population, 54.5% has access to the internet, with Europe and North America having the highest internet access rates, 85.2% and 95%, respectively [3]. In the United Kingdom, the context of this research, 94.8% of the population is connected to the internet [3], which means that the vast majority of people in the United Kingdom have the opportunity to consult the internet for health information. Indeed, many do so: the website of the UK National Health Service Choices, which offers information on symptoms, causes, and treatment for most common diseases, reportedly receives about 15 million visits per month [4].

People search for health information online to self-diagnose [5] or complement information received from the family doctor [6]. Online searching for health-related information often arises on symptom manifestation and people’s appraisal of them, known as the symptom appraisal interval (ie, the interval when people notice bodily changes, interpret them as symptoms of illness, and decide whether they warrant medical attention) [7,8]. This can be important for conditions where timeliness of presentation is an important contributor to treatment outcome. Cancer is one exemplar; prompt help-seeking for symptoms suggestive of cancer can be key to detecting cancer at an early stage and ensuring it is potentially curable [9,10]. Arguably, it is important for members of the public to have access to accurate health information online that is able to aid symptom appraisal and prompt early and appropriate help-seeking. However, this potential rests on a number of tacit assumptions which may not necessarily be met: first, that lay people experiencing bodily changes can articulate these into symptoms and into effective search terms; second, that lay people have the ability to locate online the most relevant and reliable sources of health information; and third, that, upon finding the relevant information, people can interpret it appropriately and apply it to themselves. One way to observe these processes and their implications for symptom appraisal and early symptomatic presentation is to use tools that record online information-seeking behavior in relation to a given health condition [11-13]. These tools have enriched recent research in the field of consumer online health information-seeking.

Breast cancer is one of the most frequently searched for cancer topics online [14-16], as evidenced by data from English-speaking countries including the United States, Canada, Australia and the United Kingdom [17]. Desire for online information about breast cancer appears to be affected by media coverage [18] and Breast Cancer Awareness months [19,20]. However, the high search volume may also reflect its relatively high incidence: in the United Kingdom, female breast cancer is the most common cancer among women, with around 55,000 cases diagnosed each year [21], while globally over 2 million women are diagnosed annually [22]. Indeed, it has been noted that the volume of cancer-related online searches reflects estimated cancer incidence and mortality within a given country [17,18,23], which would suggest that people are likely to seek information on breast cancer when they or someone they know receive a cancer diagnosis. However, online information sources on breast cancer can vary in their quality and completeness [24,25], and little is known about how women interpret or act on information found online in the event of breast-related symptoms.

Our previous studies on how symptomatic women make sense of symptoms indicative of breast cancer and intend to seek medical help suggested that some women do consult the internet for information about breast-related symptoms but are rather ambivalent about its value and lacking in confidence about how to appraise the information gathered [26,27]. While we have researched, in an online survey, how women attribute hypothetical breast changes to breast cancer and express preferences to seek help [28,29], we have not explored how women’s symptom attribution might translate into online information-seeking strategies or how these actions might change as a result of online searches. Therefore, in this study we extended the work we conducted in the context of breast cancer and used a browser tracking tool, Vizzata (Vizzata Limited) [30], to explore how women seek information online when presented with a symptom scenario consistent with a breast cancer symptom. We examined whether information- seeking enabled them to interpret the symptom accurately as potentially relating to breast cancer.

Another aim of our study was to determine the efficacy of a browser tracking tool to remotely capture online information-seeking behavior within a more traditional survey setting. Within the context of this study, efficacy represented the ability of the tool to record information on participant search terms and websites visited, the latter recorded as Web addresses or URLs. There are arguably two aspects to efficacy here: first, the technical ability of the tool to accurately record participant online behavior during the search task (the search terms entered and the addresses of the opened Web links). Second, the validity of the remote tool and of real-time behavior-tracking tools in general to capture behavior that can provide meaningful insights into participant reasoning processes during information-seeking.

Previous research that has captured participant online information-seeking in response to health scenarios has used, for example, screen-capture video software (eg, Camtasia, TechSmith Corporation) but often in conjunction with think-aloud tasks that documented participant reasoning during the search process [12,13]. Other studies have employed individual interviews followed by a video-recorded online search task [11,31] or face-to-face observation of the use of interactive information menus where information-seeking was operationalized as the number of links accessed [32]. Overall, these studies where interviews or think-aloud tasks accompanied or supplemented the online search have usually been conducted in face-to-face individual sessions in conditions mirroring lab settings (eg, private conference rooms, libraries, university offices) [12,13,31]. While these studies enable rich, in-depth data capture, they lack naturalism and may not necessarily mirror how people engage in online information-seeking in the context of their everyday settings (eg, at home or workplace, on a tablet or laptop). Therefore, to increase ecological validity, we used a tracking software tool that would capture remotely participant online search activities without the need for the researchers’ presence. Our approach is in line with more recent methods of capturing online information-seeking behavior, which, for example, employ internet browser extensions to log search terms and accessed website addresses [33]. In summary, our main objectives were:

1. To test the efficacy of the Vizzata browser tracking tool to capture participant online information-seeking in response to a scenario describing a hypothetical breast change (ie, a nipple rash)
2. To explore symptom attribution before and after engaging in the online search task and describe the process of online information-seeking for the hypothetical nipple rash: the search terms used and the websites accessed

## Methods

### Study Design

**Figure 1:**
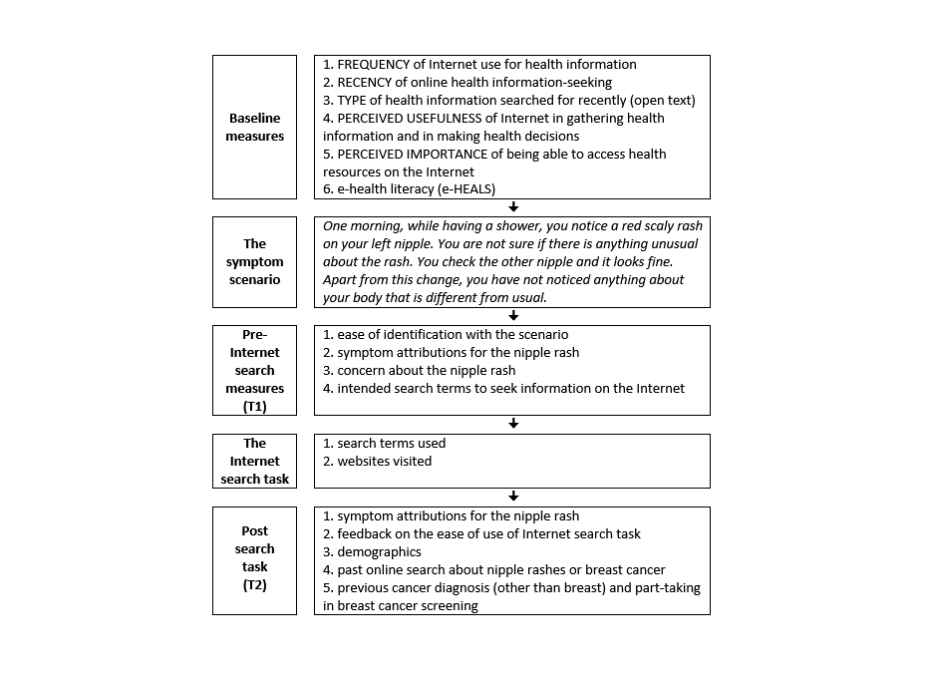
Study protocol.

The symptom scenario depicted a nipple rash—a lesser known symptom of breast cancer [34]—and was presented to participants prior to asking them to engage in the internet search task (see Figure 1).

To examine how women sought information online in relation to a hypothetical breast change indicative of breast cancer, we used the online survey platform Vizzata [30]. The browser tracking tool is an add-on feature of the Vizzata survey software and was designed to track and record search terms, URLs, and time spent on websites.

In the browser tracking section of our study, we included DuckDuckGo (duckduckgo.com) as the default search engine and instructed participants to use it within the survey internet window when performing the internet search task. DuckDuckGo looks similar to the Google search engine interface and performs similar functions and was selected because it was compatible with the browsing data capture function of the tracking tool. The key feature of DuckDuckGo is that it does not track users’ online activity. This offers a potential advantage to studies on online information-seeking as the search results during the study are less likely to be influenced by users’ past search history or other previous online activity.

### Recruitment Strategy

The study received ethical approval from the University of Surrey Ethics Committee (reference: UEC/2016/041/FHMS). A total of 125 British women were recruited via a market research company from among their online panel members between December 2016 and January 2017. The inclusion criteria were female gender, based in the United Kingdom, aged 50 years or older, with education levels ranging from no formal education to university degree or higher. The exclusion criterion was past or current breast cancer diagnosis, with the questions on breast cancer diagnosis being embedded among other health conditions (eg, diabetes) so that participants would not be aware of the focus of the study.

### Procedure

Participants were invited to the study by email invitation generated from the Vizzata platform, which informed them that the study explored how people search for health information on the internet. Figure 1 presents the study protocol followed by participants. Participants were asked to answer a few baseline questions about their use of the internet for health information before reading the symptom scenario. Then they were instructed to use the search engine to find out more about the hypothetical bodily change and to visit as many sites as they wished and then return to the final part of the survey (see Multimedia Appendix 1). A disclaimer at the start of the search task informed participants that their internet activity would be recorded only for the duration of the study and the study tracking tool would not be able to access any personal data stored in their browsers (eg, passwords).

### Materials and Measures

#### Baseline Measures

At the start of the survey (Time 1, henceforth T1), participants completed measures of frequency of internet use for health information (1 = never, 4 = very often), perceptions of the importance (1 = not important at all, 4 = very important) of the internet to provide health information, usefulness (1 = not useful at all, 4 = very useful) of the internet to provide health information and to help make decisions about one’s health, and recency of online health information-seeking (7 = this week, 6 = last week, 5 = this month, 4 = last month, 3 = a few months ago, 2 = last year, 1 = can’t remember). Participants also indicated through open text what health information they had recently searched for, which was subsequently coded as 1 if participants mentioned cancer, breast cancer, nipple, or nipple rash, 2 if participants did not mention these terms, or 3 if they couldn’t remember. We assessed eHealth literacy using the eHEALS Literacy Scale developed by Norman and Skinner [35] (Cronbach alpha = .93), who have defined eHealth literacy as “the ability to seek, find, understand, and appraise health information from electronic sources and apply the knowledge gained to addressing or solving a health problem” [36].

#### The Symptom Scenario

We included a scenario describing the appearance of a nipple rash, a largely unfamiliar symptom of breast cancer [34]. We had developed and tested the scenario with women from a range of educational backgrounds during individual cognitive think-aloud interviews (n=10) and 3 focus groups (n=19) in our past research on symptom appraisal and help-seeking intentions for breast changes among cancer-free women [28]. A subsequent online survey with 961 British women (aged 47 to 92 years) confirmed that nipple rash was a significantly less familiar symptom of breast cancer than an armpit lump [28]. We chose a relatively unfamiliar symptom to increase the variability in how women interpret and seek information for the symptom.

#### Symptom Attribution Before the Internet Search

After reading the scenario, participants completed measures assessing ease of identification with the scenario (1 = very difficult, 4 = very easy), what they thought the symptom might be (in open text), and concern about the symptom (1 = not at all, 5 = extremely). Given that search engines have predictive text (autocompletion) that can influence people’s formulation or choice of search terms, we also asked participants, prior to the start of the internet search task (T1), to indicate what search terms they intended to use in relation to the scenario.

#### The Internet Search Task

After reading the scenario, participants were required to engage in the online information-seeking task. Participants were instructed to search for information as long as they wished or until they were satisfied with the information found, and once they had finished searching they should click on the GO BACK TO SURVEY button to return to the survey and answer further questions. The data gathered during the information-seeking task included the search terms entered in the search engine and the websites visited by participants, which were saved in an Excel (Microsoft Corp) file by the Vizzata software. The viewed content was later coded yes or no as comprising breast cancer information.

#### Post Search Task

Next, participants were asked to make new symptom attributions as at T1. They also left feedback on the difficulty of the search task (1 = difficult, 2 = neither easy nor difficult, or 3 = easy) and indicated whether they had searched online for information on nipple rashes or breast cancer in the past. At the end of the survey, participants completed various demographics measures (ie, age, ethnicity, highest education level, marital status, and employment) and indicated past participation in breast cancer screening (yes/no/can’t remember/not applicable). The participants also indicated whether they had any family/friend history of breast cancer (immediate family member/other family member/close friend); these options were not mutually exclusive, and participants could check as many as applied.

## Results

### Sample Characteristics

**Figure 2:**
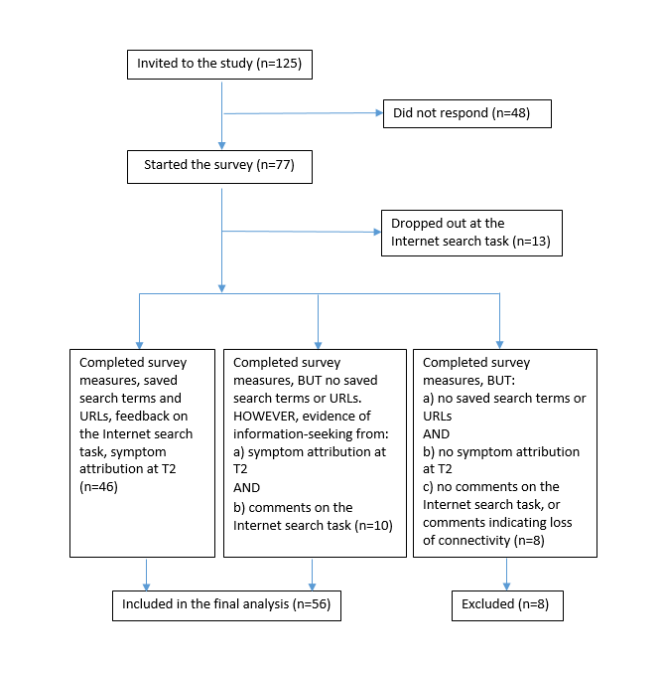
Participant recruitment and data collection flow diagram.

In total, 56 of the 125 initial panel members who were eligible and invited to the study completed the survey. Figure 2 presents a detailed description of the participants who were included and excluded from the study.

**Table 1:** Participant demographic characteristics (N=56).

Characteristics		Value, n (%)
**Highest education level**		
	No formal qualifications	12 (21)
	Education below degree level	20 (36)
	Education at degree level or above	24 (43)
**Relationship status**		
	Married/living with partner/in civil partnership	35 (63)
	Single/never married/divorced/separated/widowed	21 (38)
**Employment status**		
	Retired	26 (46)
	Employed part-time/full-time/self-employed	21 (38)
	Unemployed/homemaker/not working because of disability	9 (16)
**Ethnicity**		
	White British	54 (96)
	Other	2 (4)
**Cancer screening participation**		
	Breast cancer (eligible sample aged 50-70 years, n=49)	45 (92)
	Cervical cancer	51 (91)
	Bowel cancer (eligible sample aged 60-74 years, n=25)	16 (64)
**Family/friend history of breast cancer**		
	None	18 (32)
	Immediate family member (parent/sibling/child)	4 (7)
	Other family member	14 (25)
	Close friend	31 (55)

The majority of the women who took part in this study were White British (54/56, 96%), retired (26/56, 46%), in a relationship (35/56, 63%), and aged on average 60 years (mean 60.34 [SD 7.73] years; age range: 50-78 years). Most participants had participated in breast cancer screening (45/56, 92%), and most (38/56, 68%) had a family/friend history of breast cancer (immediate family, close friend, or a nonimmediate family member with a diagnosis of breast cancer). Summarized demographic details are presented in Table 1.

### eHealth Literacy and Recent Health Information-Seeking Online

Participants had relatively high levels of eHealth literacy (eHEALS) with regard to using the internet for health information (range: 2.13 to 5.00, median 3.88, mean 3.80, SD 0.67). The majority of the participants valued the internet as a useful source of health information (see Table 2). The majority (33/56, 59%) had searched for health information relatively recently, from last month to as recently as this week, yet none of their open-ended answers contained breast cancer or nipple rash as the focus of their recent online searches. This enabled us to assume that there would be no breast cancer search recency effects on the participants’ present searches. At the end of the study, some participants indicated they had searched in the past, although not recently, for breast cancer information online (20/56, 36%) and/or for nipple rash information (3/56, 5%).

### Efficacy of the Online Browser Tracking Tool

We assessed the efficacy of the tool (ie, its ability to capture online information-seeking behavior) by examining the number of participants who were able to complete the internet search task and the participant feedback on the browser tracking tool as an embedded element of the survey.

**Table 2:** Participant baseline use of the internet for health information (N=56).

Characteristics		Value, n (%)	
**Frequency of internet use to find information about health**			
	Occasionally		45 (80)
	Often or very often		10 (18)
	Never		1 (2)
**The last time internet was used for health information**			
	This week/last week/this month/last month		33 (59)
	A few months ago/last year		21 (38)
	Can’t remember		2 (4)
**Past online searches for nipple rash**			
	Yes		3 (5)
	No		52 (93)
	Can’t remember		1 (2)
**Past online searches for breast cancer**			
	Yes		20 (36)
	No		36 (64)
**Internet useful to gather health information**			
	Very useful		14 (25)
	Useful		41 (73)
	Not useful		1 (2)
**Internet useful to help make decisions about health**			
	Very useful		5 (9)
	Useful		47 (84)
	Not useful		4 (7)
**Important to access health resources on the internet**			
	Very important		17 (30)
	Important		31 (55)
	Not important		8 (14)

#### Data Captured and Missing Data

As described in the flow diagram (Figure 2), 83% (64/77) of responders completed the survey. For 28% (18/64) of those, no search terms or visited websites (URLs) were saved during the internet search task. We inspected the incomplete data of these respondents to understand reasons for noncompletion and assess whether participants had engaged in information-seeking without this being captured; we compared the symptom attribution before (T1) and after (T2) the internet search task and examined their feedback on the internet browser tracking element. Eight of these 18 participants did not complete the internet search or had connectivity problems, as indicated by their comments (eg, “none of the sites would open” and “I was putting it into the correct space but nothing was happening?”). These 8 participants did not provide any information after the internet search task that would have indicated successful engagement in information-seeking, therefore we excluded them from further analyses.

However, 10 participants of the 18 whose data on search terms and URLs were missing were retained for further analyses because they provided feedback on the information-seeking task and sufficiently meaningful data for comparison of symptom attribution between T1 and T2, which indicated they had indeed engaged in online information-seeking despite this not being captured. We included these participants in the final sample because the focus of our study was not solely on what websites the participants visited but also on how the process of searching for health information on the internet had influenced symptom appraisal and symptom attribution at T2. One participant, for example, whose search terms or URLs were not recorded, reported finding the information on the National Health Service (NHS) site useful, particularly about Paget disease, and made symptom attributions at T2 that included cancer (in contrast to T1): “Eczema. Insect bite. Psoriasis. Paget disease – breast cancer. Mastitis.”

The missing data on search terms and URLs and the feedback of these 10 participants whose data were incomplete yet meaningful suggest that some may have misunderstood the task instructions (eg, installed the DuckDuckGo search engine and conducted searches in separate windows away from that of the survey), as one participant’s feedback indicates:

Downloading search engine a bit tricky. Search itself easy.

Other participants may have had problems with the internet connection:

...kept losing connection to search engine.

In other cases the participants may have used internet browsers other than the recommended Google Chrome, which may have been incompatible with the Vizzata tracking tool, or may have used devices other than PC/laptop, which may have reduced the survey usability:

I struggled to find the “go back to survey” button as I’m using my phone.

#### Feedback on the Search Task

Overall, participants left positive feedback on the search task. When returning to the survey, the participants left comments as to whether the internet search task had been easy to do. We coded the open-ended responses of the participants into easy (47/56, 84%), difficult (2/56, 4%), and neither easy nor difficult (7/56, 13%). The feedback suggests that the majority of the participants did not encounter significant problems with the DuckDuckGo search engine or with the Vizzata tracking tool and that any missing data pertaining to the search terms or the accessed websites may have been due to reasons other than participant-related factors. Some participants encountered but overcame technical issues, such as slow internet connection:

Yes [easy], just a bit slow.

...sometimes the search engine said oops problem in searching but on the whole it came up with lots of different sites to use for more research.

### Symptom Attribution

#### Symptom Attribution and Related Measures at T1

Most participants who completed the survey (48/56, 86%) found the scenario fairly or very easy to imagine themselves in, and most (34/56, 61%) indicated that they would be only a little bit or moderately concerned about the nipple rash.

At both T1 and T2, we coded the symptom attributions as “cancer,” “environmental,” “physical,” or “don’t know” in line with our previous research [28]. We coded as missing attribution those responses that contained neither a symptom attribution nor a “don’t know” response (eg, “a symptom worth further investigation”). If a participant made multiple attributions in their responses (eg, “an ordinary rash, an allergic reaction, cancer”), we coded each attribution separately. The majority of participants (30/56, 54%) made a single symptom attribution, and just under half (25/56, 45%) made a cancer attribution. Attributing the symptom to physical causes (eg, eczema) was common at T1 (32/56, 57%).

#### Intended Search Terms

We coded the intended search terms as 1 = breast or skin cancer, 2 = nipple rash or rash on nipples, 3 = rash on skin/breast, 4 = other skin-related conditions, and 5 = nipple or breast changes. One “not sure” response was coded as missing data. The most frequently intended search terms were rash-related phrases (nipple rash, nipple red rash, red scaly rash on nipple), mentioned by 86% of the participants (48/56). Only 3 participants intended to use breast cancer and 1 participant, skin cancer on the breast (all had made a cancer attribution at T1).

#### Search Terms Used

The actual search terms were coded as follows: 1 = breast or skin cancer, 2 = nipple rash or rash on nipples, 3 = rash on breast, 4 = nipple or breast change, and 5 = other. Only 2 participants (2/46, 4%) included cancer in their search terms. Of the participants who had made a cancer attribution at T1, 4% (1/25) used cancer in their search terms. The majority (40/46, 87%) used rash-related search terms, particularly nipple rash and rash on nipple (see Table 3 for a list of all the search terms used). It is noteworthy that while the scenario specified “a red scaly rash on your left nipple,” some participants (3/56, 5%) added sensory terms (eg, itchy) which were not in the original scenario; this could have influenced the returned results and thus their interpretation of the symptom.

A very small number of participants (5/46, 11%) changed their search terms during the internet search task, which reflects their interpretation of the information found and their reappraisal of the symptom in light of the information. For example, participant #57 started with “changes in nipples,” changed to “breast cancer symptoms,” and then more specifically “breast cancer symptoms NHS.” Participant #50 started with “red scaly rash on nipple” and later changed to “What are the chances of red scaly rash on nipple being cancer?” Other participants changed their search terms by focusing on the physical aspects of the hypothetical symptom (eg, from “red scaly rash on nipple” to “itchy rash on nipple” [participant #70]) or made their searches less specific, changing from “scaly rash on nipple” to “scaly rash” (participant #142). However, the vast majority of participants (41/46, 89%) did not change their search terms during the search task.

### Websites Visited

A total of 9% (5/46) of the complete cases did not go beyond the first page with search results returned by the search engine. For these participants, the number of websites visited was coded as zero but not as missing data because these participants were nonetheless able to view some preview of the search results and thus be exposed to some information rather than none at all. The returned results pages included Web links mentioning Paget disease of the nipple or links to information on breast cancer. Three of the five participants who did not go beyond the first search results page changed from not suspecting cancer at T1 to making a cancer attribution at T2, which supports the view that information exposure can have an effect.

Overall, the number of websites visited ranged from 0 to 6 (mean 1.96, SD 1.30, median 2, mode 1). We coded the content viewed by the participants as containing cancer-related content (yes/no) (eg, information on breast cancer or Paget disease of the breast). The majority of participants whose browsing data were saved (41/46, 89%) viewed websites containing breast cancer information, in particular Paget disease (see Table 4 with a list of the websites visited). Excluding the 10 participants whose Web browsing data were not saved, there was no significant association between making a cancer attribution at T1 and accessing cancer-specific websites (*χ*^2^_1,46_=0.004, *P*=.95. Furthermore, the participants who made a cancer attribution at T1 did not visit more websites than those who did not make a cancer attribution at T1 (*t*_1,44_=0.647, *P*=.52).

**Table 3:** The range of search terms used (N=46).

Search terms used	Participants using search term, n (%)
Red scaly rash on nipple	9 (20)
Nipple rash	7 (15)
Breast rash	2 (4)
Itchy rash on nipple	2 (4)
Rash on nipple	2 (4)
Scaly rash on nipple	2 (4)
Ask NHS^a^ questions	1 (2)
Breast cancer symptoms	1 (2)
Breast cancer symptoms NHS	1 (2)
Breast changes	1 (2)
Breast health red scaly rash	1 (2)
Changes in nipples	1 (2)
Itchy red breast rash	1 (2)
Nipple rash scaly	1 (2)
Nipple red rash	1 (2)
Rash on breast area	1 (2)
Rash on female breast	1 (2)
Rash on nipple area	1 (2)
Rash on nipples problems	1 (2)
Red breast rashes	1 (2)
Red rash nipple	1 (2)
Red rash on nipple	1 (2)
Red scaly nipple rash	1 (2)
Red scaly rash	1 (2)
Red scaly rash around nipple	1 (2)
Red scaly rash in nipple	1 (2)
Red scaly rash on breast	1 (2)
Red scaly rash on left nipple	1 (2)
Scaley rash on nipples *(sic)*	1 (2)
Scaly rash	1 (2)
Scaly rash on nipple NHS	1 (2)
Scaly red nipple rash	1 (2)
Scaly red rash on nipple	1 (2)
Sore nipples	1 (2)
What are the chances of red scaly rash on nipple being cancer	1 (2)
What should I do if I suspect I have itchy nipples	1 (2)

^a^NHS: National Health Service.

**Table 4:** The range of websites (domains) visited (n=41).

Web domains visited	Breast cancer content (yes/no)	Participants visiting the website, n (%)
www.nhs.uk	Yes	21 (51)
www.skinsight.com	Yes	8 (20)
www.rightdiagnosis.com	Yes	7 (17)
www.webmd.boots.com	Yes	7 (17)
symptoms.rightdiagnosis.com	No	6 (15)
www.healthcentral.com	Yes	4 (10)
www.mayoclinic.org	Yes	4 (10)
healthunlocked.com	No	3 (7)
www.breastcancer.org	Yes	3 (7)
www.zocdoc.com	No	3 (7)
www.cancerresearchuk.org	Yes	2 (5)
www.healthhype.com	Yes	2 (5)
www.healthline.com	Yes	2 (5)
about-cancer.cancerresearchuk.org	Yes	1 (2)
breastcancernow.org	Yes	1 (2)
en.m.wikipedia.org	Yes	1 (2)
en.wikipedia.org	Yes	1 (2)
healthguides.healthgrades.com/treating-psoriatic-arthritis/	No	1 (2)
www.cancer.gov	Yes	1 (2)
www.everydayhealth.com	Yes	1 (2)
www.macmillan.org.uk	Yes	1 (2)
www.medhelp.org	Yes	1 (2)
www.nationalbreastcancer.org	Yes	1 (2)
www.phaa.com	Yes	1 (2)
www.webmd.com	No	1 (2)

**Table 5:** Attributions for the nipple rash at T1 and T2 (n=56).

Type of attribution	T1 (before internet search task), n (%)	T2 (after internet search task), n (%)
Cancer	25 (45)	37 (66)
Physical	26 (46)	32 (57)
Environmental	16 (29)	3 (5)
Don’t know	7 (13)	4 (7)
Missing attribution	2 (4)	2 (4)

### Symptom Attribution at T2

After viewing information online, the majority (31/56, 55%) of participants made a single symptom attribution at T2. More participants made a cancer attribution at T2 (37/56, 66%) compared to T1 (25/56, 45%), although some participants changed from making a cancer attribution at T1 to a noncancer one at T2 (6/56, 11%). There was a noticeable increase in medical terms used to make symptom attributions at T2 compared to T1, showing the exposure to formal medical terms during the online search: Paget disease (22 vs 1), eczema (14 vs 8), dermatitis (12 vs 3), and mastitis (3 vs 0) (see Table 5 for a summary of symptom attributions at T1 and T2). There was no significant association between viewing websites with breast cancer content and making a cancer attribution at T2 (*χ*^2^_1,46_=1.92, *P*=.31).

## Discussion

### Principal Findings

Regarding the efficacy of the browser tracking tool to capture online information-seeking, the results are rather mixed and point to challenges in measuring information-seeking remotely and designing reliable and user-friendly tools. In the majority of cases, the Vizzata tracking tool captured the search terms participants entered in the search engine and the websites they accessed, yet there were instances where participants seemed to encounter technical problems. Close inspection of the incomplete data of the 18 participants, of which 10 were retained in the study, enabled us to envisage a number of reasons why the search and browsing data were not saved. First, it is possible that some participants may have misunderstood the task instructions and installed the DuckDuckGo search engine, thus conducting the search within a browsing window outside that of the survey and preventing their online behavior from being recorded. Unfamiliarity with the DuckDuckGo search engine or with internet search tasks may have contributed to this. These potential errors on the part of participants suggest that online tracking tools need to be intuitive and user-friendly in order to be used effectively without the researcher’s facilitation. Using a training session with a mock search prior to the actual study to familiarize the participants with the search task, as other researchers have done, could be a useful way to safeguard against misunderstandings or technical problems [12,13].

Second, it is possible that, despite the instructions, some participants may have used other internet browsers than the recommended Google Chrome that may have had features incompatible with the Vizzata tracking tool (eg, blocking tracking tools by default). Third, the tracking tool itself may not be sensitive enough to deal with the software complexities required to record online searching and browsing. However, we cannot make a direct comparison of the Vizzata tracking tool and other methods used in similar studies (eg, the Camtasia screen capture video-recording software [12,13]), and it is likely that each software package or method has its own advantages and disadvantages [37]. As has been noted before, researchers aiming to capture online behavior need to be realistic about the capabilities and limitations of each tool and decide on their use according to factors such as ease of participant recruitment, naturalistic setting, data accuracy, and need to minimize technical complexity [37,38].

Furthermore, the failure to capture some participants’ online searching behavior (either for participant- or software-related reasons) also points to the limits of recording participant online behavior remotely. Our research had a limited scope of testing the capabilities of the Vizzata tracking tool ahead of a larger survey. The results suggest that more sophisticated study designs with additional measures (eg, think-aloud tasks or individual interviews) may be necessary to produce a more accurate and detailed picture of online health information-seeking behavior. A number of researchers have noted that capturing naturalistic online information-seeking behavior is a complex process that can be challenging [33,37,38], and it may well be that facing technical problems (delay in loading webpages or slow speed of custom tools that track online behavior) is not unusual in this type of study [38]. For example, researchers conducting a think-aloud task in conjunction with a search task using either the Google search engine or WebMD’s Symptom Checker reported that participants found it difficult to navigate the programs and troubleshoot them after receiving error messages [11]. Further challenges noted in the literature relate to developing adequate tools that can record online behavior remotely such as search terms, visualization of Web pages, scrolling through search engine results pages, time spent on different Web pages, and gathering, where necessary, enough contextual information to enrich the data collected remotely and help interpret it [38].

Regarding symptom attribution, at T1 less than half of the participants (25/56) attributed the symptom (red scaly rash on nipple) to cancer, which supports the finding that nipple rash is an unfamiliar symptom of breast cancer [28,34]. While 25 participants thought the symptom could be cancer, only 1 of these used cancer in their search terms. The fact that not all cancer attributions at T1 translated into cancer-related search terms suggests that the participants’ searches were inductive (ie, driven by symptoms) rather than deductive (ie, driven by diagnosis assumptions such as cancer). The results suggest that suspecting cancer at T1 did not necessarily translate into a hypothesis-testing approach to health information-seeking as found in other studies [12]. In line with similar research on online health information-seeking in response to symptoms, the participants’ search strategies suggest an evidence-gathering approach to information-seeking rather than a hypothesis-testing one [12].

During the search task, some participants did not go beyond the first page of results, a behavior observed in other studies of health information-seeking [13]. However, the majority of participants accessed websites containing breast cancer information, in particular Paget disease of the breast. Half of these participants viewed the webpage of the UK NHS presenting information on Paget disease. Yet, not all participants interpreted the nipple rash as a symptom of breast cancer despite coming across information linking the symptom to the disease. Nonetheless, seeking online information did have an impact, as 12 more participants made a cancer attribution at T2 compared to T1. Some participants maintained their cancer attribution from T1, while others changed from noncancer at T1 to cancer at T2, although a small number of participants also changed from making a cancer attribution at T1 to a noncancer one at T2.

However, cancer attributions made in light of information- seeking should not be viewed as categorical interpretations of the symptom but rather as a possibility among many. While some participants made a single attribution at T2, others made more than one attribution as they envisaged the possibility that the symptom could be eczema or other physical condition (eg, anything from just a rash to something really serious like a sign of cancer [participant #70]). Importantly, our previous research [28] found that attributing nipple rash to cancer (as at least one possible cause) was associated with increased likelihood of medical help-seeking, which is an important behavior for earlier diagnosis of cancer [9].

### Limitations

This study was conducted on a relatively small sample of participants (n=56) which limits the generalizability of our findings. However, small samples are not unusual in surveys that aim to capture online information-seeking behavior. Sample sizes in these studies vary from 20 [31] to 54 [33] and 78 [12].

The majority of participants did not refine their search terms or consult many websites. As noted in other studies [31], the hypothetical nature of the symptom scenario may have demotivated participants to sustain in-depth information-seeking. It could well be that when people are acutely experiencing troubling symptoms they may be more thorough in their seeking and interpretation of online health information.

Another limitation of this study is that we were not able to explore how the participants made sense of the information found and what made them change (or not) their symptom attribution after engaging in information-seeking. Exploring how people engage with the information found online (eg, what websites seem trustworthy and why) could have provided more insight into how the participants interpreted online health information in relation to the given symptom. As has been pointed out in similar research, examining online health information-seeking requires attending to the cognitive and perceptual processes that are involved in conducting and interpreting an internet search [11].

Last, it is noteworthy that participants’ search terms closely mirrored the symptom described in the scenario, “red scaly rash on nipple” and “nipple rash” being the most frequently used ones. This could be due to the fact that the symptom scenario was presented in text that made the symptom explicit. It is possible that had the symptom been presented in visual rather than textual form the participants may have interpreted it differently and used a wider range of search terms. Further research in the field of online health information-seeking is needed to explore how symptom attribution varies according to textual versus visual scenarios.

### Conclusions

This exploratory study revealed that, despite some technical limitations, it is possible to capture the process of online information-seeking in relation to possible cancer symptoms. This work has potential for impact, both in terms of developing methodology to understand real-world issues and furthering the research agenda on understanding responses to cancer symptoms and engagement in health information-seeking online.

## Appendix

Multimedia Appendix 1 Screenshot of instructions on how to conduct the information-seeking task.

## Abbreviations

NHS: National Health Service
