# Supplementary material for: Online Information-Seeking About Potential Breast Cancer Symptoms: Capturing Online Behavior With an Internet Browsing Tracking Tool
Source: J Med Internet Res. 2019 Feb 6;21(2):e12400. doi: 10.2196/12400 (PMC6381403; doi:10.2196/12400)
Supplement: Multimedia Appendix 1 [file jmir_v21i2e12400_app1.pdf]

Multimedia Appendix 1

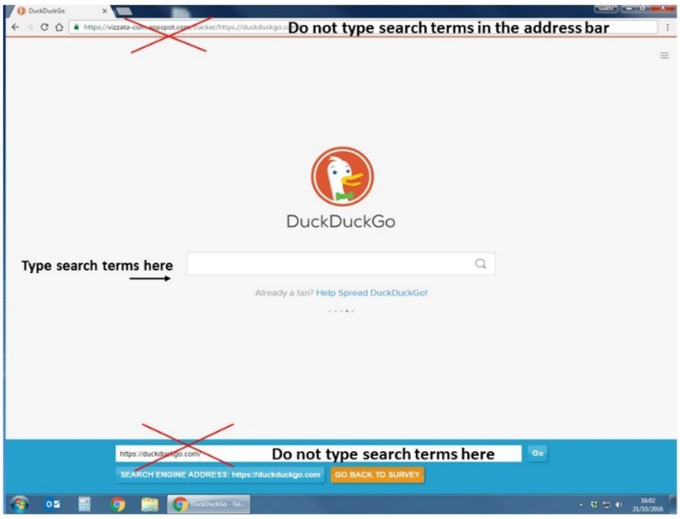

Screenshot of instructions on how to conduct the information-seeking task
